# Workflow for Genome-Wide Determination of Pre-mRNA Splicing Efficiency from Yeast RNA-seq Data

**DOI:** 10.1155/2016/4783841

**Published:** 2016-12-06

**Authors:** Martin Převorovský, Martina Hálová, Kateřina Abrhámová, Jiří Libus, Petr Folk

**Affiliations:** Department of Cell Biology, Faculty of Science, Charles University, Viničná 7, 128 43 Prague 2, Czech Republic

## Abstract

Pre-mRNA splicing represents an important regulatory layer of eukaryotic gene expression. In the simple budding yeast* Saccharomyces cerevisiae*, about one-third of all mRNA molecules undergo splicing, and splicing efficiency is tightly regulated, for example, during meiotic differentiation.* S. cerevisiae* features a streamlined, evolutionarily highly conserved splicing machinery and serves as a favourite model for studies of various aspects of splicing. RNA-seq represents a robust, versatile, and affordable technique for transcriptome interrogation, which can also be used to study splicing efficiency. However, convenient bioinformatics tools for the analysis of splicing efficiency from yeast RNA-seq data are lacking. We present a complete workflow for the calculation of genome-wide splicing efficiency in* S. cerevisiae* using strand-specific RNA-seq data. Our pipeline takes sequencing reads in the FASTQ format and provides splicing efficiency values for the 5′ and 3′ splice junctions of each intron. The pipeline is based on up-to-date open-source software tools and requires very limited input from the user. We provide all relevant scripts in a ready-to-use form. We demonstrate the functionality of the workflow using RNA-seq datasets from three spliceosome mutants. The workflow should prove useful for studies of yeast splicing mutants or of regulated splicing, for example, under specific growth conditions.

## 1. Introduction

In eukaryotes, coding parts of genes, the exons, are interrupted by noncoding parts, the introns. The process through which introns are removed and exons are joined together is called splicing. It occurs via two consecutive transesterification reactions which are catalysed by the spliceosome, a large dynamic ribonucleoprotein complex composed of five snRNP particles (U1, U2, U4/U6, and U5) and other associated protein complexes, like the Nineteen Complex (NTC in yeast; CDC5L in mammals) (reviewed in [1]). Splicing must occur very precisely as even a single nucleotide shift may lead to a frameshift, which could cause many disorders, including cancer [2, 3]. Therefore, regulation of splicing has an important role in gene expression.

Introns are defined by core sequences comprising the 5′ splice site, branch site, and the 3′ splice site. In metazoans, additional sequences are needed for recruiting various* trans*-acting regulatory factors, which modulate the binding of spliceosome subunits and splice site choice and efficiency, deciding on the splicing outcome. This is important especially for alternative splicing (reviewed in [4]).

In contrast to higher eukaryotes, whose genes typically contain multiple short exons alternating with introns up to several kilobases long [5], gene structure in the budding yeast* Saccharomyces cerevisiae* is much simpler. Only five percent of the almost 6000 yeast genes contain introns, usually just one [6]. However, the intron containing genes are very highly expressed. As a result, about one-third of all transcripts are spliced [7]. The consensus sequences of the yeast core spliceosome signals are well defined (GUAUGU for the 5′ splice site, UACUA**A**C for the branch site with the branching A in bold, and AG for the 3′ splice site) [6]. Also, there are only few cases of alternative splicing in budding yeast (reviewed in [8]), and regulation of splicing efficiency plays a more prominent role, for example, during meiosis [9] or under environmental stress [10, 11]. For example, the constitutively transcribed intron containing genes* REC107*,* AMA1*,* SPO22*, and* MER3* are efficiently spliced and processed to form functional mRNAs only during meiosis, when the Mer1 splicing factor is expressed [9, 12–14]. Also, various environmental stresses can lead to differential changes in splicing efficiency in specific groups of genes: amino acid starvation inhibits splicing of the ribosomal protein genes, while ethanol stress has no effect on this group of genes but alters splicing in another group [10, 11].

The yeast spliceosome consists of ~90 proteins, that is, around half of the proteins identified in the human spliceosome. However, nearly all of the yeast spliceosome components have counterparts in human. Therefore, it was suggested that the* S. cerevisiae* spliceosome represents an evolutionarily conserved core of the splicing machinery. Accordingly, many of the human-specific spliceosomal proteins are needed for the regulation of alternative splicing, a feature almost missing in the budding yeast [15]. This, together with the ease of cultivation and genetic manipulation, led to the establishment of the budding yeast as a favourite model for studying the basic mechanisms of pre-mRNA splicing.

To study the influence of genetic perturbations or environmental conditions on splicing, it is important to quantify the splicing efficiency. Splicing efficiency is traditionally calculated as the amount of mRNA divided by the amount of pre-mRNA. The gold standard for mRNA and pre-mRNA quantification is the use of quantitative PCR (RT-qPCR) with primers spanning exon-intron and exon-exon junctions (e.g., [16]). However, this approach is feasible for measuring mRNA and pre-mRNA levels for only a limited number of genes. By contrast, ultrahigh-throughput sequencing of RNA (RNA-seq) allows comprehensive splicing analysis at the genome-wide scale [17–19]. There are multiple paradigms for calculating splicing efficiency from RNA-seq data, which are based on comparing sequencing read counts from intronic and exonic regions or also take into account exon-exon junction reads (transreads). The methods also vary in the length of the window considered (e.g., 25 bp around a splice site versus a whole exon) [17, 18, 20–24]. RNA-seq is a simple, robust, and affordable technique and there is now a wealth of publicly available RNA-seq datasets [25, 26]. Together, this makes RNA-seq a convenient method for genome-wide determination of splicing efficiency, although the bioinformatics analyses involved might be challenging for nonspecialists. Here, we present a complete and documented up-to-date workflow for semiautomatic calculation of genome-wide splicing efficiencies from strand-specific RNA-seq data in* S. cerevisiae*.

## 2. Materials and Methods

### 2.1. RNA-seq Datasets

Sequencing reads from strand-specific transcriptome profiling of splicing mutants (*prp45*(1-169),* prp4-1*, and* prp40-1*) and their corresponding wild-type* S. cerevisiae* strains [17, 24] were downloaded from the European Nucleotide Archive (http://www.ebi.ac.uk/ena) in FASTQ format (more information about the various file formats used in this study can be found at https://genome.ucsc.edu/FAQ/FAQformat.html) and experiment metadata were obtained from ArrayExpress (https://www.eabi.ac.uk/arrayexpress/). The relevant database accession numbers are given in Table 1. Importantly, to simplify downstream analyses, only “read 1” FASTQ files were used from paired-end sequencing datasets.

The pipeline requires that strand-specific sequencing read data are used (true for most currently generated RNA-seq datasets). As pervasive antisense transcription has been reported in many eukaryotic organisms, including yeasts [27], strand specificity of sequencing reads helps to separate the contributions of potential overlapping sense/antisense transcripts.

Sequencing read quality and potential contamination by adapters and/or PCR primers was checked with FastQC 0.11.5 (http://www.bioinformatics.babraham.ac.uk/projects/fastqc/). All datasets were found suitable for further analyses. However, if needed, contaminating and low-quality sequences can be filtered and/or trimmed using tools such as Trimmomatic [28].

### 2.2. Read Mapping

Reads were aligned to* S. cerevisiae* genome (Ensembl R64-1-1) with the fast, splice-aware HISAT2 aligner (version 2.0.4) using* S. cerevisiae* genome index containing transcript structures [29]. Minimum and maximum intron length parameters were set to 20 and 10000 nt, respectively. More details on HISAT2 parameter settings can be found in the “workflow.sh” shell script in the Supplementary Material available online at http://dx.doi.org/10.1155/2016/4783841.

Subsequently, samtools 1.3.1 were used to filter reads by their mapping quality score (MAPQ ≥ 10) to keep only reads that aligned unambiguously to a single locus and to sort and index the resulting BAM files [30]. Read mapping was also assessed visually in the IGV browser 2.3.69 [31].

We also tried aligning reads from the* PRP45*-related datasets with TopHat2 [32], the widely used predecessor of HISAT2. Compared to TopHat2, the HISAT2 alignment step was ~2-fold faster, and the mapping supported identification of ~11% more transreads and calculation of splicing efficiencies for ~4% more splice sites.

### 2.3. Splice Site Identification and Counting of Transreads

Putative splicing events were detected by regtools 0.2.0 (https://regtools.readthedocs.io/en/latest/). These tools look for presumed transreads (reads spanning exon-exon junction) in BAM files, compile a table of all identified putative splice sites and their characteristics, and provide the counts of transreads spanning these splice sites. Minimum and maximum intron length parameters were set to 20 and 10000 nt, respectively. Detected splice sites were also annotated and classified as known or novel using regtools and* S. cerevisiae* genome annotation (Ensembl R64-1-1) in GTF format. The output from regtools was further processed and coordinates of bases at the very 5′ and 3′ ends of each known intron were extracted (into BED format) by a custom R script (R version 3.3.1, https://www.r-project.org/). See the “workflow.sh” and “junctions.R” scripts in the Supplementary Material for more details.

### 2.4. Determination of 5′ and 3′ Intron End Coverage

Read coverage of the very first (5′ end) and the very last (3′ end) base of each known intron was determined from all BAM files in parallel using bedtools 2.25.0 (bedtools multicov) [33]. It is critical to set the* -split* parameter to avoid including transreads in the intronic read counts. It is also important to correctly set the* -s*/*-S* parameters according to the sequencing library preparation protocol employed to ensure that only reads mapped to the “sense” strand will be counted (see the “workflow.sh” script in the Supplementary Material).

### 2.5. Splicing Efficiency Calculation

The method for splicing efficiency calculation was derived from the “3′ splice site ratio” method described in [20]. For each intron, splicing efficiency was determined separately for the 5′ splice site and 3′ splice site using the following formulas:

(1) Efficiency  5′=transread  count5′  intron  end  first  base  coverage,Efficiency  3′=transread  count3′  intron  end  last  base  coverage.

Transreads only arise from spliced transcripts and thus reflect directly the abundance of mRNA molecules in which the particular intron has been spliced out. By definition, transreads must cover at least the very last base of exon X and the very first base of exon X + 1. To match this single-base resolution while counting intronic reads, only those reads covering the very first (for 5′ splice site) and the very last (for 3′ splice site) base of the corresponding intron are counted. This allows direct comparison of pre-mRNA and spliced mRNA molecule levels (an approach most similar to the RT-qPCR gold standard; see below) and separate calculation of splicing efficiencies at the 5′ splice site and 3′ splice site of each intron, reflecting the efficiencies of the two splicing steps.

We note that, for some genes, our method may use only a fraction of all available intronic reads (e.g., for mitochondrial genes with long introns). Importantly, the introns of some genes harbour nested snoRNAs or are predicted to contain potential stable structures, suggesting the presence of so far uncharacterised nested noncoding RNAs. It was shown that such introns can be maintained in the cell after splicing [34], potentially affecting the determination of splicing efficiency when sequencing reads along the whole intron are considered.

Depending on the sequencing library size, strain genotype, or cultivation conditions, many introns typically have very low read coverage, which might produce unreliable splicing efficiency data. Therefore, in the final spreadsheet, we do not report splicing efficiency values for junctions with read counts below an arbitrary threshold of 5 reads for both transread count and intron end read count. Users may change this threshold if required; see the “efficiency.R” script in the Supplementary Material for more details.

### 2.6. Yeast Strains and Cell Cultivation

Wild-type (BY4741-MATa* his3*Δ*1 leu2*Δ*0 met15*Δ*0 ura3*Δ*0*) and the* prp45*(1-169) mutant (AVY17-MATa* his3*Δ*1 leu2*Δ*0 met15*Δ*0 ura3*Δ*0 prp45*(1-169)-3-HA::NatMX6) strains were grown in the YPAD medium (2% peptone, 1% yeast extract, 0.01% adenine, and 2% glucose) at 30°C to 1.5 × 10^7^ cells/mL. Two millilitres of each culture was harvested by centrifugation and cell pellets were stored at −80°C.

### 2.7. RNA Isolation, Reverse Transcription, and RT-qPCR Analysis

Total RNA was isolated with the MasterPure Yeast RNA Isolation Kit (Epicentre) according to the manufacturer's protocol. cDNA was prepared from 2 *μ*g of the total RNA using the RevertAid First Strand cDNA Synthesis Kit (Thermo Fisher Scientific) with random hexamer primers. RT-qPCR was performed using LightCycler 480 II (Roche). Each reaction (total volume of 10 *μ*L) was performed in triplicate and consisted of 5 *μ*L of the MESA GREEN qPCR MasterMix Plus for SYBR Assay, No-ROX (Eurogentec), 4 *μ*L of 100-fold diluted cDNA, and a pair of primers 0.3 mM each (for sequences, see the Supplementary Material). Primer pairs were designed to specifically amplify either the spliced or the unspliced transcripts of the* ECM33*,* ACT1*,* COF1*,* RPL22A*, and* RPL22B* genes. Amplicons from unspliced transcripts covered the 3′ splice junction in* ECM33*, while the 5′ splice junction was covered in the other four genes. Four to six biological replicates were analysed for each gene, and the* TOM22* and* SPT15* genes were used as reference controls. Relative pre-mRNA and mRNA quantities were calculated by the ΔΔCt method [35].

## 3. Results and Discussion

### 3.1. Pipeline for Determination of Splicing Efficiency

**Figure 1:**
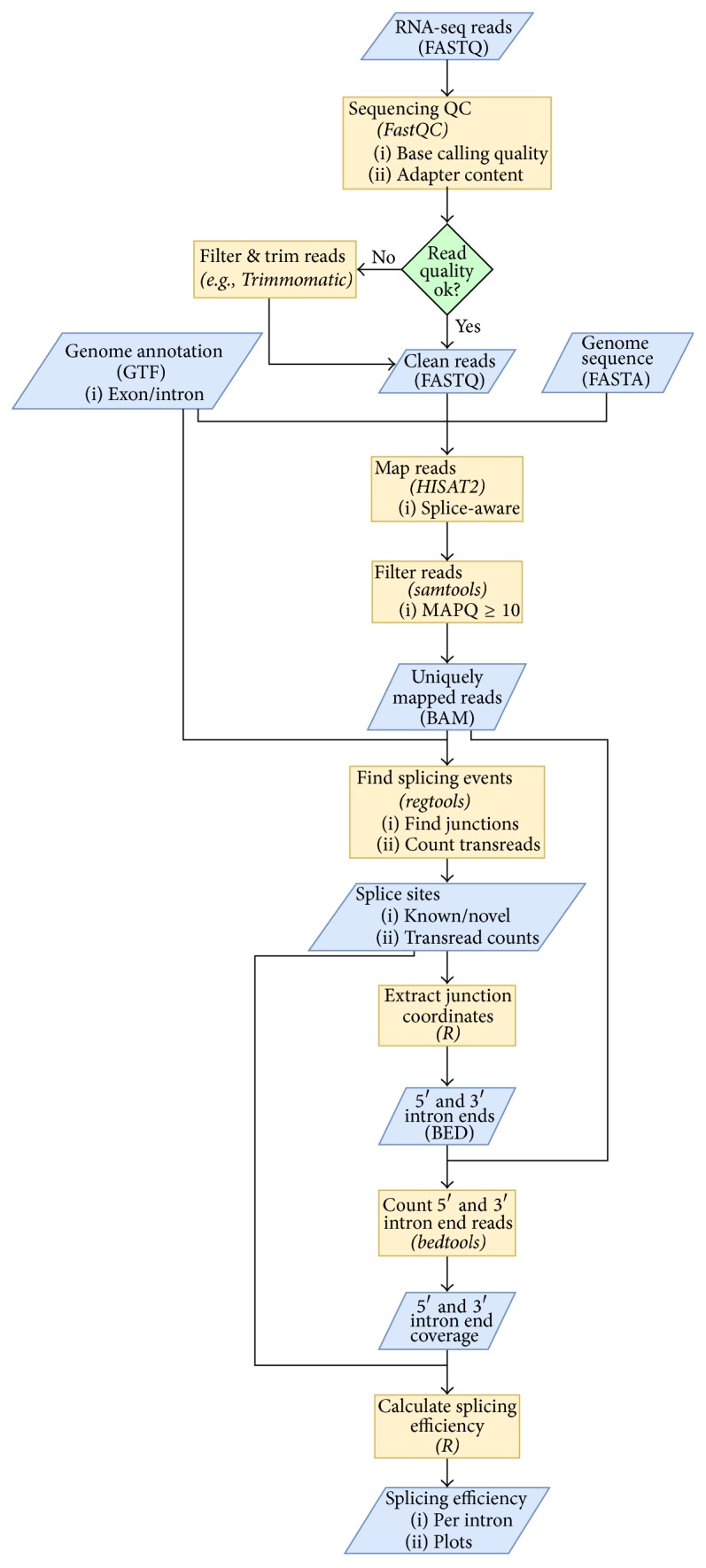
Workflow for calculating splicing efficiency from RNA-seq data. Files and datasets are represented by blue parallelograms (file formats given in parentheses), and processing steps are represented by orange rectangles (tool names given in parentheses). Some files/datasets are used repeatedly in several steps of the workflow as signified by multiple flow lines going from these files/datasets. The diagram was created using draw.io (https://www.draw.io/).

We have put together a workflow for calculating splicing efficiency of each intron from standard RNA-seq data in* S. cerevisiae* (Figure 1). The pipeline consists mostly of established open-source tools for manipulation and analysis of next-generation sequencing data (FastQC, HISAT2, samtools, regtools, and bedtools) and simple custom scripts (Linux shell and R). All scripts including parameter settings for all tools are available in the Supplementary Material.

Briefly, after input quality control (FastQC), reads are mapped into* S. cerevisiae* reference genome (HISAT2, [29]) and filtered, keeping only uniquely mapped reads (samtools, [30]; MAPQ ≥ 10). Positions of splice junctions (both known and novel) and transread counts for these junctions are extracted (regtools). Read coverage of the 5′ and 3′ intron end base is determined (bedtools, [33]) and splicing efficiency calculated for each intron at both 5′ and 3′ splice junction, corresponding to the efficiency of the first and the second step of splicing, respectively.

The description of folder structure and content, both required before starting the analysis and produced during the analysis, is given in the file “folders_readme” in the Supplementary Material. Users need to supply genome sequence (in FASTA format), annotation (in GTF format), and HISAT2 genome index containing transcript structures (in the “genome” folder). A ready-made* S. cerevisiae *genome index can be downloaded from the HISAT2 website (“genome_tran”; https://ccb.jhu.edu/software/hisat2/). Users further need to supply RNA-seq reads in FASTQ format (in the “FASTQ” folder); the pipeline is designed for single-end, strand-specific sequencing data. Depending on the protocol for sequencing library preparation that was used, users might need to adjust strandness-related parameters for HISAT2 and bedtools (see the Materials and Methods and the “workflow.sh” file in the Supplementary Material) to obtain correct read counts and splicing efficiency values.

The main outputs of the pipeline are

1. a table (CSV format) of transread counts from all samples for known splice junctions (folder “transreads”; file “splice_junctions_coverage.known.csv”; note that only junctions of expressed genes for which transreads were detected are reported in all analyses);
2. tables of read coverage from all samples for 5′ and 3′ terminal bases of known introns (folder “introns”; files “introns_known_5ss.bed.counts.csv” and “introns_known_3ss.bed.counts.csv”);
3. tables of read threshold-filtered splicing efficiencies in all samples calculated separately for 5′ and 3′ splice sites of known introns (folder “efficiency”; files “splicing_efficiency_5ss_conf.csv” and “splicing_efficiency_3ss_conf.csv”).

**Figure 2:**
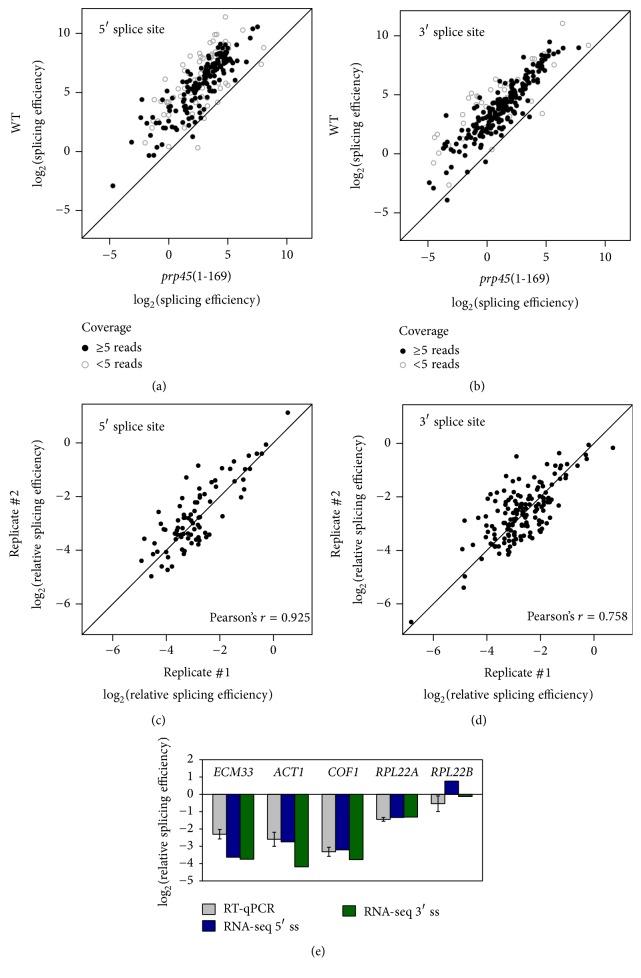
Splicing efficiency in the* prp45*(1-169) mutant. Splicing efficiencies for all known introns (for 5′ and 3′ splice sites separately) were calculated using the pipeline described in Figure 1. (a, b) Results for two pooled biological replicates of the* prp45*(1-169) mutant and its corresponding wild-type strain. Higher values correspond to more efficient splicing. Full circles represent values for introns with sufficient coverage (≥5 transreads and ≥5 reads covering intron end base); open circles represent low-confidence values for introns with low sequencing read coverage. (c, d) Relative splicing efficiencies (*prp45*(1-169) normalized to wild type) at the 5′ and 3′ splice sites were calculated for each biological replicate separately. Only introns with sufficient read coverage were considered. Pearson's *r* values for the two replicates are indicated. (e) Comparison of relative splicing efficiencies at the 5′ and 3′ splice sites of selected genes calculated from the pooled RNA-seq data with relative splicing efficiencies determined by RT-qPCR (means of 4–6 independent RT-qPCR experiments ± SD).

Users can also define a set of sample (FASTQ filename) pairs to be compared (e.g., mutant versus wild type; see the “efficiency.R” script in the Supplementary Material). The pipeline then produces a table of “relative” splicing efficiencies (folder “efficiency”; files “relative_splicing_efficiency_5ss_conf.csv” and “relative_splicing_efficiency_3ss_conf.csv”) and scatterplot images (folder “images”; PDF format; see Figure 2 for an example).

Multiple approaches have been used in the past to determine splicing efficiency from yeast RNA-seq data, for example, [17, 18, 21, 24]. However, the bioinformatics tools used are often outdated now in terms of speed of processing and their abilities to work with split reads (transreads). Also, the complete workflow, including all relevant scripts, is usually not provided. By contrast, the pipeline presented in this study is based upon the latest tools with advanced capabilities for fast and accurate split read processing, suitable for studies of splicing efficiency [29, 30, 33]. The pipeline also allows convenient processing of multiple FASTQ files (samples) with very limited input from the user required. In case of the* PRP45*-related datasets mentioned below, ~55 million 100 nt reads were processed by the pipeline in 38 minutes on a standard desktop PC (quad-core AMD A8-3870 APU CPU with 8 GB RAM) running 64-bit Ubuntu 16.04.

While the scripts provided in the Supplementary Material are customized for analysis of* S. cerevisiae* RNA-seq data, the pipeline can be easily adapted for other yeast species by providing the corresponding genome sequence, genome annotation, and genome index (built using hisat2-build [29]) files and altering the relevant filename variables in the “workflow.sh” script accordingly. The Supplementary Material contains one such example adaptation of the pipeline for the fission yeast* Schizosaccharomyces pombe*.

### 3.2. Analysis of Splicing Efficiency in Spliceosome Mutants

As we are primarily interested in studying altered splicing patterns in various spliceosome mutants, we selected three publicly available RNA-seq datasets for* S. cerevisiae* spliceosome mutants, which show global reductions in splicing efficiency, to demonstrate the function of our workflow. All datasets contain samples with roughly similar amounts of sequencing reads (~17–35 million) of very similar length (~100 nt) but differ markedly in data quality in terms of the percentage of uniquely mappable reads (Table 1).

The first example dataset is focused on Prp45, an essential splicing factor and a component of the so-called NTC-related complex [36]. Based on cryo-EM structural information, Prp45 stabilizes the catalytic centre of the spliceosome through interactions with many proteins and with all three spliceosomal snRNAs [37]. Cells bearing the C-terminally truncated* prp45*(1-169) allele are temperature-sensitive and have deformed shapes [38]. We analysed two biological replicates of RNA-seq data for wild-type and* prp45*(1-169) cells grown at permissive temperature (30°C). First, we used the data pooled by genotype and found a global decrease of splicing efficiency in the mutant (Figures 2(a) and 2(b)). Next, we calculated relative splicing efficiencies (i.e., mutant normalized to wild type) in each replicate separately to assess reproducibility. Results for introns with sufficient splice junctions coverage (≥5 transreads and ≥5 reads covering intron end base) showed good agreement between the two independent replicates (Figures 2(c) and 2(d)). Finally, to validate the results of our pipeline, we used RT-qPCR to measure the levels of spliced and unspliced transcripts for five selected genes showing various degrees of splicing impairment in the* prp45*(1-169) mutant RNA-seq dataset. Reassuringly, the splicing efficiencies calculated by our pipeline were concordant with those determined by RT-qPCR, pointing to possible differential requirements for Prp45 in pre-mRNA splicing of specific genes (Figure 2(e)).

**Figure 3:**
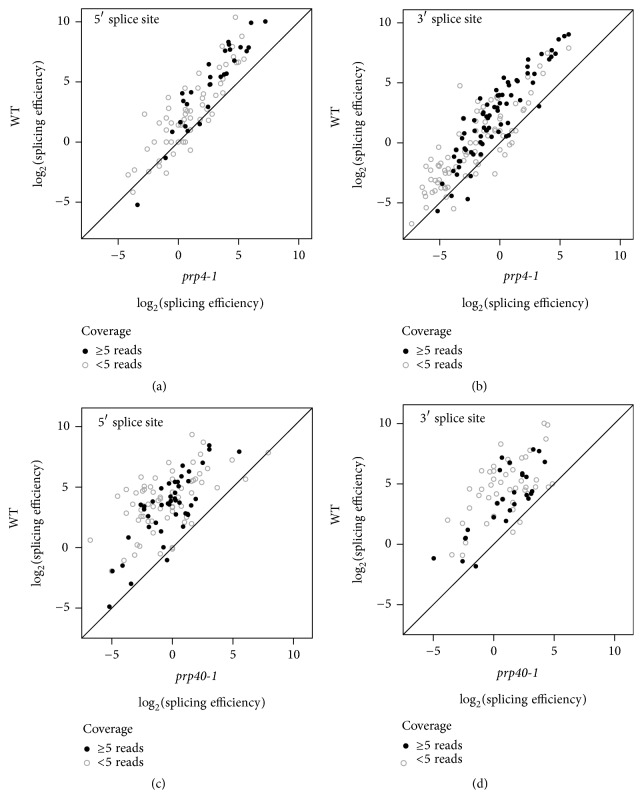
Splicing efficiency in the* prp4-1* and* prp40-1* mutants. Splicing efficiencies for all known introns (for 5′ and 3′ splice sites separately) were calculated using the pipeline described in Figure 1. (a, b) Results for the* prp4-1* mutant and its corresponding wild-type strain [17]. (c, d) Results for the* prp40-1* mutant and its corresponding wild-type strain [24]. Higher values correspond to more efficient splicing. Full circles represent values for introns with sufficient coverage (≥5 transreads and ≥5 reads covering intron end base); open circles represent low-confidence values for introns with low sequencing read coverage.

Next, we analysed data for Prp4, a structural component of the U4/U6 di- and U4/U6-U5 tri-snRNP particles [39, 40]. The* prp4-1* temperature-sensitive allele blocks U4 snRNP dissociation during the catalytic activation of the spliceosome [41, 42]. Using splicing-sensitive microarrays, it was shown that this mutation causes a genome-wide splicing defect even at the permissive temperature of 26°C [43]. We used the* prp4-1* RNA-seq dataset from [17] and we confirmed the global impairment of splicing efficiency in this mutant at permissive temperature (Figures 3(a) and 3(b)). However, for a number of introns, splicing efficiency could not be convincingly determined (especially at the 5′ splice site) due to low read coverage at the splice junctions. This was unexpected since the* prp4-1* sequencing library sizes and mappability were comparable to the* prp45*(1-169) dataset (Table 1). Furthermore, the whole distribution of splicing efficiencies at the 3′ splice site (for both* prp4-1* and its wild-type control) was shifted compared to the other two splicing mutants we analysed. Visual inspection of mapped* prp4-1* reads revealed decreasing coverage towards 5′ ends of genes, where introns are typically located in* S. cerevisiae*, suggesting possible problems with RNA sample preparation and/or processing.

The third example relates to Prp40, a component of the U1 snRNP particle [44]. Through interactions with many spliceosome subunits and with the phosphorylated C-terminal domain of the RNA polymerase II, the Prp40 protein is important for cotranscriptional spliceosome assembly (reviewed in [45]). The* prp40-1* temperature-sensitive mutant [44] exerts a global splicing defect when shifted to nonpermissive temperature [24], which we confirmed by running the published RNA-seq dataset through our pipeline (Figures 3(c) and 3(d)). It should be noted that this dataset had poor mappability (Table 1) and yielded relatively low read coverage, decreasing the number of junctions for which splicing efficiency could be calculated convincingly.

Thus, these three proof-of-principle scenarios demonstrated that our workflow is able to recapitulate previously identified genome-wide decreases in splicing efficiency using RNA-seq data. The results also highlight a critical requirement for sufficient sequencing library quality and size for successful analysis.

## 4. Conclusions

We present a complete bioinformatics workflow for determining splicing efficiency in the budding yeast* S. cerevisiae* using data produced by the simple and affordable RNA-seq technique. Starting with strand-specific sequencing reads in the FASTQ format, our pipeline is able to calculate splicing efficiency at the 5′ and 3′ splice junctions of each intron, with very limited input required from the user. All relevant scripts are provided in a documented and ready-to-use form. The workflow should prove useful for studies of yeast splicing mutants or of regulated splicing, for example, under various growth conditions.

## Supplementary Material

The Supplementary Material contains a table of PCR primers used in this study, and all scripts required to run the analyses of splicing efficiency presented in Figures 2 and 3. The scripts are commented and can be easily adapted for other RNA-seq datasets. An adaptation of the workflow for processing RNA-seq data from the fission yeast Schizosaccharomyces pombe is also included.

## Figures and Tables

**Table 1:** RNA-seq datasets used in this study.

Genotype	ArrayExpress acc. number^a^	ENA acc. number^b^	Read length (nt)	Total reads	Reads with MAPQ ≥ 10	% reads with MAPQ ≥ 10
WT	E-MTAB-5149	ERR1709739	100	27 789 829	25 329 092	91,2%
		ERR1709740	100	22 000 062	20 402 556	92,7%
*prp45*(1-169)	E-MTAB-5149	ERR1709737	100	27 842 215	25 491 566	91,6%
		ERR1709738	100	25 156 639	23 359 541	92,9%
WT	E-GEOD-44219	SRX233529	100	21 012 048	17 127 536	81,5%
*prp4-1*	E-GEOD-44219	SRX233535	100	17 142 559	14 457 015	84,3%
WT	E-GEOD-49966	SRR953535	101	35 203 753	7 655 225	21,8%
*prp40-1*	E-GEOD-49966	SRR953537	101	17 326 529	3 596 304	20,8%

^a^Accession number for the ArrayExpress database (https://www.ebi.ac.uk/arrayexpress/).

^b^Accession number for the European Nucleotide Archive (http://www.ebi.ac.uk/ena).

## References

[1] Will C. L., Lührmann R. (2011). Spliceosome structure and function. *Cold Spring Harbor Perspectives in Biology*.

[2] Ward A. J., Cooper T. A. (2010). The pathobiology of splicing. *Journal of Pathology*.

[3] Singh R. K., Cooper T. A. (2012). Pre-mRNA splicing in disease and therapeutics. *Trends in Molecular Medicine*.

[4] Wang Z., Burge C. B. (2008). Splicing regulation: from a parts list of regulatory elements to an integrated splicing code. *RNA*.

[5] Deutsch M., Long M. (1999). Intron-exon structures of eukaryotic model organisms. *Nucleic Acids Research*.

[6] Spingola M., Grate L., Haussler D., Manuel A. (1999). Genome-wide bioinformatic and molecular analysis of introns in Saccharomyces cerevisiae. *RNA*.

[7] Ares M., Grate L., Pauling M. H. (1999). A handful of intron-containing genes produces the lion's share of yeast mRNA. *RNA*.

[8] Kempken F. (2013). Alternative splicing in ascomycetes. *Applied Microbiology and Biotechnology*.

[9] Engebrecht J., Voelkel-Meiman K., Roeder G. S. (1991). Meiosis-specific RNA splicing in yeast. *Cell*.

[10] Bergkessel M., Whitworth G. B., Guthrie C. (2011). Diverse environmental stresses elicit distinct responses at the level of pre-mRNA processing in yeast. *RNA*.

[11] Pleiss J. A., Whitworth G. B., Bergkessel M., Guthrie C. (2007). Rapid, transcript-specific changes in splicing in response to environmental stress. *Molecular Cell*.

[12] Nakagawa T., Ogawa H. (1999). The Saccharomyces cerevisiae MER3 gene, encoding a novel helicase-like protein, is required for crossover control in meiosis. *EMBO Journal*.

[13] Munding E. M., Igel A. H., Shiue L., Dorighi K. M., Treviño L. R., Ares M. (2010). Integration of a splicing regulatory network within the meiotic gene expression program of *Saccharomyces cerevisiae*. *Genes & Development*.

[14] Davis C. A., Grate L., Spingola M., Ares M. (2000). Test of intron predictions reveals novel splice sites, alternatively spliced mRNAs and new introns in meiotically regulated genes of yeast. *Nucleic Acids Research*.

[15] Fabrizio P., Dannenberg J., Dube P. (2009). The evolutionarily conserved core design of the catalytic activation step of the yeast spliceosome. *Molecular Cell*.

[16] Hao S., Baltimore D. (2013). RNA splicing regulates the temporal order of TNF-induced gene expression. *Proceedings of the National Academy of Sciences of the United States of America*.

[17] Munding E. M., Shiue L., Katzman S., Donohue J. P., Ares M. (2013). Competition between pre-mRNAs for the splicing machinery drives global regulation of splicing. *Molecular Cell*.

[18] Kawashima T., Douglass S., Gabunilas J., Pellegrini M., Chanfreau G. F. (2014). Widespread use of non-productive alternative splice sites in saccharomyces cerevisiae. *PLoS Genetics*.

[19] Bitton D. A., Rallis C., Jeffares D. C. (2014). LaSSO, a strategy for genome-wide mapping of intronic lariats and branch points using RNA-seq. *Genome Research*.

[20] Herzel L., Neugebauer K. M. (2015). Quantification of co-transcriptional splicing from RNA-Seq data. *Methods*.

[21] Livesay S. B., Collier S. E., Bitton D. A., Bähler J., Ohi M. D. (2013). Structural and functional characterization of the N terminus of *Schizosaccharomyces pombe* Cwf10. *Eukaryotic Cell*.

[22] Grisdale C. J., Bowers L. C., Didier E. S., Fast N. M. (2013). Transcriptome analysis of the parasite Encephalitozoon cuniculi: an in-depth examination of pre-mRNA splicing in a reduced eukaryote. *BMC Genomics*.

[23] Gould G. M., Paggi J. M., Guo Y. (2016). Identification of new branch points and unconventional introns in Saccharomyces cerevisiae. *RNA*.

[24] Volanakis A., Passoni M., Hector R. D. (2013). Spliceosome-mediated decay (SMD) regulates expression of nonintronic genes in budding yeast. *Genes and Development*.

[25] Ozsolak F., Milos P. M. (2011). RNA sequencing: advances, challenges and opportunities. *Nature Reviews Genetics*.

[26] Reuter J. A., Spacek D. V., Snyder M. P. (2015). High-throughput sequencing technologies. *Molecular Cell*.

[27] Jensen T. H., Jacquier A., Libri D. (2013). Dealing with pervasive transcription. *Molecular Cell*.

[28] Bolger A. M., Lohse M., Usadel B. (2014). Trimmomatic: a flexible trimmer for Illumina sequence data. *Bioinformatics*.

[29] Kim D., Langmead B., Salzberg S. L. (2015). HISAT: a fast spliced aligner with low memory requirements. *Nature Methods*.

[30] Li H., Handsaker B., Wysoker A. (2009). The sequence alignment/map format and SAMtools. *Bioinformatics*.

[31] Thorvaldsdóttir H., Robinson J. T., Mesirov J. P. (2013). Integrative Genomics Viewer (IGV): high-performance genomics data visualization and exploration. *Briefings in Bioinformatics*.

[32] Kim D., Pertea G., Trapnell C., Pimentel H., Kelley R., Salzberg S. L. (2013). TopHat2: accurate alignment of transcriptomes in the presence of insertions, deletions and gene fusions. *Genome Biology*.

[33] Quinlan A. R., Hall I. M. (2010). BEDTools: a flexible suite of utilities for comparing genomic features. *Bioinformatics*.

[34] Hooks K. B., Naseeb S., Parker S., Griffiths-Jones S., Delneri D. (2016). Novel intronic RNA structures contribute to maintenance of phenotype in Saccharomyces cerevisiae. *Genetics*.

[35] Schmittgen T. D., Livak K. J. (2008). Analyzing real-time PCR data by the comparative *C*
_T_ method. *Nature Protocols*.

[36] Ohi M. D., Link A. J., Ren L., Jennings J. L., McDonald W. H., Gould K. L. (2002). Proteomics analysis reveals stable multiprotein complexes in both fission and budding yeasts containing Myb-related Cdc5p/Cef1p, novel pre-mRNA splicing factors, and snRNAs. *Molecular and Cellular Biology*.

[37] Wan R., Yan C., Bai R., Huang G., Shi Y. (2016). Structure of a yeast catalytic step I spliceosome at 3.4 Å resolution. *Science*.

[38] Gahura O., Abrhámová K., Skružný M. (2009). Prp45 affects Prp22 partition in spliceosomal complexes and splicing efficiency of non-consensus substrates. *Journal of Cellular Biochemistry*.

[39] Banroques J., Abelson J. N. (1989). PRP4: a protein of the yeast U4/U6 small nuclear ribonucleoprotein particle. *Molecular and Cellular Biology*.

[40] Bjørn S. P., Soltyk A., Beggs J. D., Friesen J. D. (1989). PRP4 (RNA4) from *Saccharomyces cerevisiae*: its gene product is associated with the U4/U6 small nuclear ribonucleoprotein particle. *Molecular and Cellular Biology*.

[41] Hartwell L. H., McLaughlin C. S., Warner J. R. (1970). Identification of ten genes that control ribosome formation in yeast. *MGG Molecular & General Genetics*.

[42] Ayadi L., Miller M., Banroques J. (1997). Mutations within the yeast U4/U6 snRNP protein Prp4 affect a late stage of spliceosome assembly. *RNA*.

[43] Clark T. A., Sugnet C. W., Ares M. (2002). Genomewide analysis of mRNA processing in yeast using splicing-specific microarrays. *Science*.

[44] Kao H.-Y., Siliciano P. G. (1996). Identification of Prp40, a novel essential yeast splicing factor associated with the U1 small nuclear ribonucleoprotein particle. *Molecular and Cellular Biology*.

[45] Becerra S., Andrés-León E., Prieto-Sánchez S., Hernández-Munain C., Suñé C. (2016). Prp40 and early events in splice site definition. *Wiley Interdisciplinary Reviews: RNA*.

